# A Hidden Diagnosis: Neurogenic Bladder Leading to Acute Kidney Failure in Wolfram Syndrome

**DOI:** 10.7759/cureus.83594

**Published:** 2025-05-06

**Authors:** Nadia Echcharii, Nabila Chekhlabi, Amal Haoudar, Nezha Dini

**Affiliations:** 1 Pediatrics, Cheikh Khalifa International University Hospital, Mohammed VI University of Health Sciences (UM6SS), Casablanca, MAR; 2 Anesthesiology, Cheikh Khalifa International University Hospital, Mohammed VI University of Health Sciences (UM6SS), Casablanca, MAR

**Keywords:** acute kidney failure, genetic analysis, multisystemic manifestations, neurogenic bladder, wolfram syndrome

## Abstract

Wolfram syndrome (WS) is a rare, autosomal recessive neurodegenerative disorder characterized by progressive multisystemic involvement, including diabetes mellitus, optic atrophy, diabetes insipidus, sensorineural hearing loss, and urological dysfunction. While diabetes mellitus is typically the first clinical sign, atypical presentations can delay the diagnosis.

We report the case of a 15-year-old boy, born to consanguineous parents, who presented to the Emergency Department with status epilepticus due to acute kidney failure. His renal impairment resulted from urinary retention caused by undiagnosed neurogenic bladder dysfunction. His medical history included type 1 diabetes mellitus diagnosed at age 5, bilateral optic atrophy at age 9, and persistent polyuria and polydipsia, later diagnosed as central diabetes insipidus. Additional findings included bilateral sensorineural hearing loss and brain MRI abnormalities. Genetic testing confirmed a homozygous pathogenic mutation in the WFS1 gene, establishing the diagnosis of WS.

This case underscores the importance of recognizing urological manifestations in WS, as neurogenic bladder dysfunction can lead to severe renal complications. WS should be considered in patients with a combination of diabetes mellitus, optic atrophy, and unexplained urinary symptoms, particularly in consanguineous populations. Early recognition and multidisciplinary management are crucial to preventing life-threatening complications and improving patient outcomes.

## Introduction

Wolfram syndrome (WS), first described in 1938, is a rare autosomal recessive disorder characterized by progressive neurodegeneration affecting multiple systems. The clinical spectrum includes diabetes mellitus, optic atrophy, diabetes insipidus, hearing loss, and urological dysfunction [1]. Early diagnosis is crucial to preventing severe complications such as renal failure secondary to urinary dysfunction [2]. WS is classified into two subtypes: WS type 1 (WS1), caused by mutations in the WFS1 gene on chromosome 4p16, and WS type 2 (WS2), associated with CISD2 mutations on chromosome 4q22. WS1 is typically more severe and carries a poor prognosis [3]. The prevalence of WS is estimated to range from one in 60,000 to 770,000 individuals worldwide [4]. Most patients experience premature mortality due to severe neurological complications, including bulbar dysfunction and organic brain syndrome [2].

Here, we report a case of WS where acute kidney failure due to neurogenic bladder dysfunction was the initial presenting feature. This atypical manifestation highlights the importance of considering WS in patients with unexplained multisystemic symptoms.

## Case presentation

**Figure 1:**
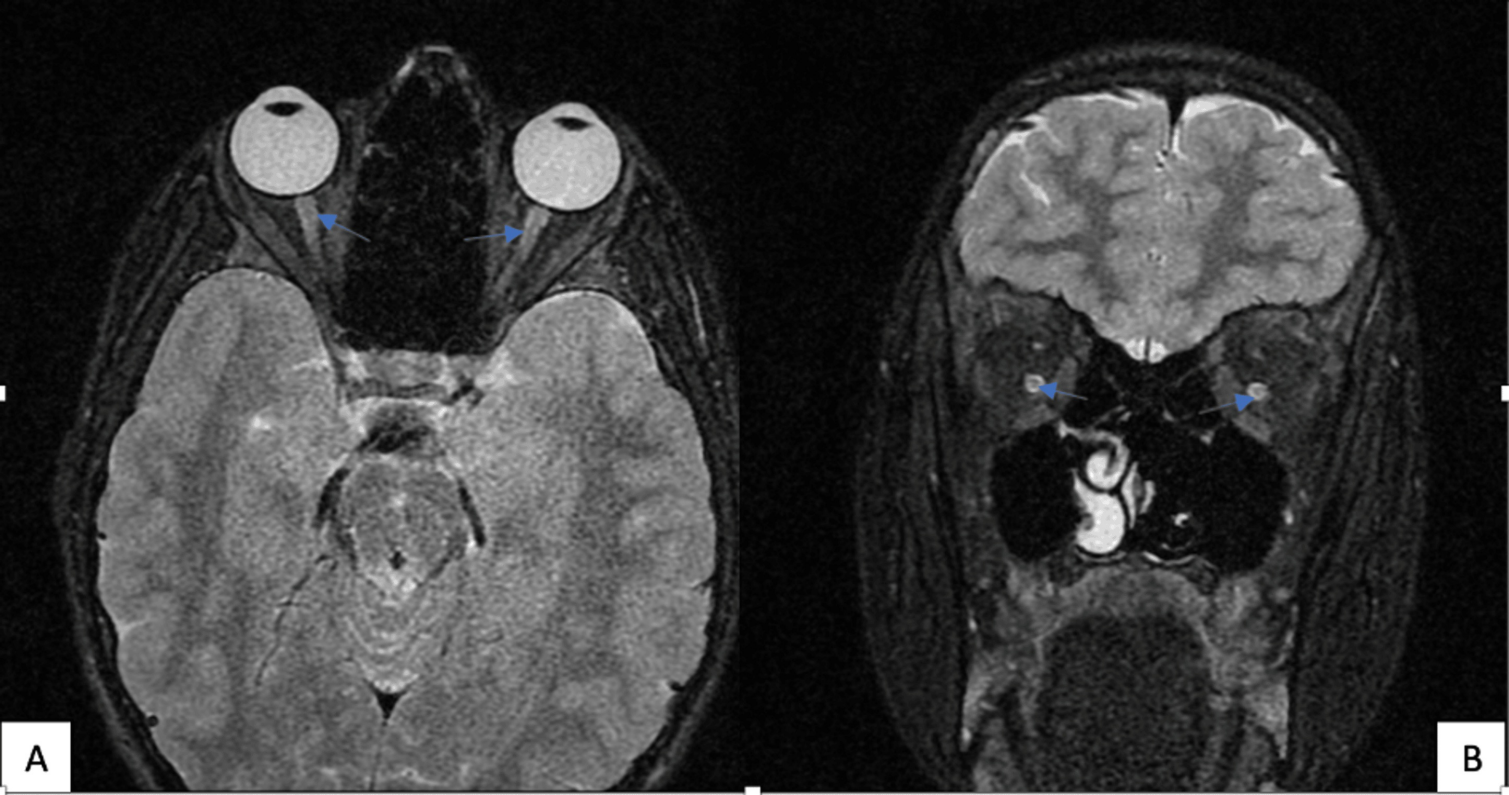
Bilateral optic atrophy in a patient with Wolfram syndrome (A and B) MRI showing symmetrical optic nerve atrophy (disk pallor) in axial and coronal views

A 15-year-old boy, born to consanguineous parents, presented with status epilepticus due to hyperuricemia secondary to acute kidney failure. Renal impairment was caused by undiagnosed neurogenic bladder dysfunction leading to urinary retention. This was supported by markedly elevated serum creatinine levels, which reflected obstructive acute kidney injury caused by urinary retention, later confirmed by urodynamic evaluation. His medical history revealed type 1 diabetes mellitus diagnosed at age 5, progressive bilateral optic atrophy (Figure 1) at age 9, and recurrent episodes of acute pyelonephritis. Two months prior, he had been hospitalized for acute pyelonephritis, with an ultrasound revealing bilateral ureterohydronephrosis. Urinary symptoms included urgency, secondary enuresis, and two prior episodes of acute urinary retention. Additionally, he reported persistent polyuria and polydipsia, which had not been further investigated. Family history revealed paternal relatives affected by type 1 and type 2 diabetes.

On admission, he was unconscious, agitated, pale, dehydrated, and afebrile, with a normal capillary blood glucose level (0.94 g/dL), and was hypertensive (196/110 mmHg), tachycardic (140 bpm), and tachypneic (35 breaths/min), with generalized edema, particularly in the eyelids, along with urinary incontinence. Urine tests indicated hematuria (2+), proteinuria (1+), and positive urine glucose without ketones. He was administered diazepam followed by a loading dose of phenobarbital.

Laboratory tests confirmed acute kidney failure (urea: 18.3 mmol/L, creatinine: 643.4 μmol/L), severe metabolic acidosis (pH 7.02, serum bicarbonate of 12 mmol/L), hyperkalemia (7 mEq/L), hypernatremia (153 mEq/L), and an HbA1c of 15.8%. Liver function was normal. Hematological analysis showed a white blood cell count of 25,800/μL with predominant neutrophils (21,000/μL), hemoglobin level of 11 g/dL, and C-reactive protein (CRP) level of 2.82 mg/dL (Table 1).

**Table 1:** Main biological parameters at admission CRP: C-reactive protein

Parameter	Value	Normal range
Urea	18.3 mmol/L	2.5-7.5 mmol/L
Creatinine	643.4 μmol/L	50-120 μmol/L
Sodium	153 mEq/L	135-145 mEq/L
Potassium	7 mEq/L	3.5-5 mEq/L
Glucose	159 mg/dL	70-110 mg/dL
HbA1c	15.8%	<6.5%
Blood pH	7.02	7.35-7.45
HCO_3_	2 mmol/L	22-26 mmol/L
White blood cells	25,800/μL	4,000-10,000/μL
Hemoglobin	11 g/dL	12-16 g/dL
CRP	2.82 mg/dL	<5 mg/dL

The elevated white blood cell count and CRP level were consistent with an inflammatory response, likely related to a urinary tract infection, in the context of recent acute pyelonephritis. He required urgent hemodialysis, leading to a significant improvement in renal function.

**Figure 2:**
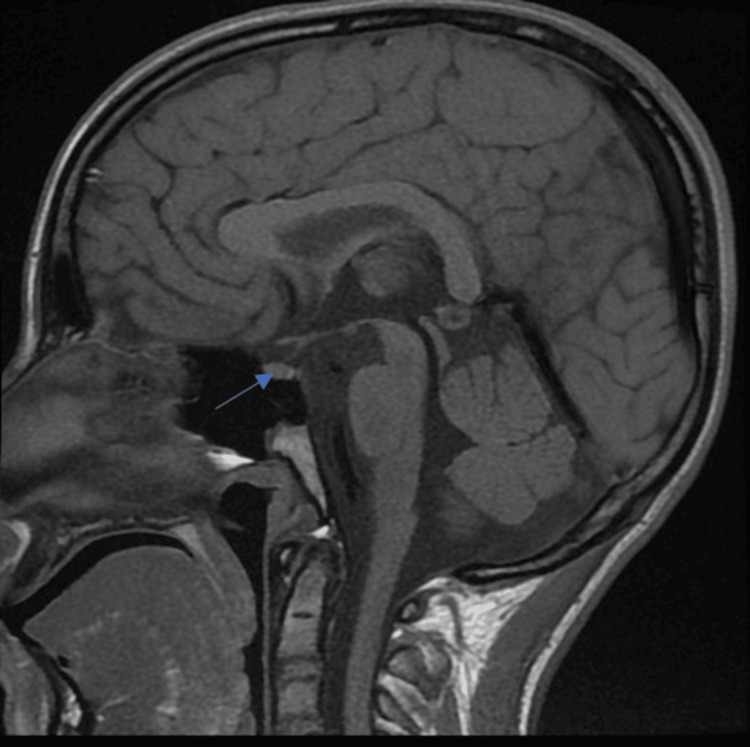
Brain MRI showing posterior pituitary signal loss, confirming central diabetes insipidus in a patient with Wolfram syndrome

During hospitalization, persistent polyuria and polydipsia, associated with hypernatremia, despite normal blood glucose levels, prompted further investigations. A water deprivation test followed by desmopressin administration confirmed complete central diabetes insipidus. Brain MRI revealed the absence of the posterior pituitary bright spot on T1-weighted images (Figure 2), reinforcing the diagnosis. Audiometry demonstrated bilateral sensorineural hearing loss of 40 dB in the right ear and a mixed hearing loss with a predominance of sensorineural component of 35 dB in the left ear.

Ophthalmologic evaluation confirmed severe visual impairment with visual acuity of 2/10 in both eyes. In addition, investigations revealed other manifestations. These included bilateral ureterohydronephrosis, without reflux on cystography; EEG showed numerous bursts of generalized and synchronous spike-waves and poly spike-waves. Furthermore, a urodynamic study showed vesicosphincteric dyssynergia with bladder hyperactivity, increasing the risk of upper urinary tract complications. Self-catheterization was initiated.

Given the combination of type 1 diabetes, optic atrophy, central diabetes insipidus, neurogenic bladder dysfunction, and hearing loss, WS was suspected. Genetic analysis confirmed a homozygous pathogenic mutation in the WFS1 gene. The patient also exhibited psychological distress, requiring psychiatric care and antidepressant therapy.

This case highlights an unusual presentation of WS, where acute kidney failure due to neurogenic bladder dysfunction was the initial reason for medical consultation. It underscores the importance of considering WS in patients with a combination of diabetes mellitus, optic atrophy, and unexplained urinary dysfunction, particularly in consanguineous populations. Early recognition and multidisciplinary management are crucial to preventing severe complications and improving patient outcomes.

## Discussion

WS, also known as DIDMOAD (diabetes insipidus, diabetes mellitus, optic atrophy, and deafness), is a rare autosomal recessive neurodegenerative disorder primarily caused by mutations in the WFS1 gene, though a less common form (WS2) results from mutations in CISD2 [4]. WFS1 encodes wolframin, a protein crucial for calcium homeostasis and endoplasmic reticulum function. Its deficiency leads to cellular stress, early apoptosis, and progressive dysfunction of metabolically active tissues, particularly pancreatic β-cells, neurons, and the optic nerve. In contrast, WFS2 mutations are associated with additional features such as bleeding disorders and gastrointestinal ulcers [5,6].

Our case illustrates an atypical presentation of WS1, with acute kidney failure secondary to acute urinary retention. This renal involvement as an initial manifestation highlights the need to consider WS in patients with unexplained multisystemic symptoms. While diabetes mellitus is typically the first clinical sign, appearing between ages 6 and 10, it is often followed by progressive optic atrophy leading to severe visual impairment [7,8]. In our patient, these classic features were present, but the worsening clinical picture due to severe urological dysfunction, including vesicosphincteric dyssynergia and bilateral hydronephrosis, is unusual.

Urological complications in WS, particularly neurogenic bladder dysfunction, are frequent but often underdiagnosed. If not managed early, they can lead to recurrent urinary tract infections, urinary tract dilation, and ultimately, severe renal impairment. Regular urological assessments, including urodynamic studies and renal ultrasound, are essential for early detection and intervention. Systematic screening and a multidisciplinary approach, including intermittent self-catheterization and medical therapy, are crucial to preserving renal function, preventing long-term complications, and improving patient outcomes [9-11].

Our patient also exhibited sensorineural hearing loss, confirmed by audiometry, another classical feature of WS. This progressive impairment often necessitates hearing aids or cochlear implants. Additionally, neurological abnormalities such as seizures and psychiatric disorders, as seen in our case, further contribute to the complexity of the disease and require a tailored neuropsychiatric approach [2].

The diagnosis of WS relies on the presence of these characteristic clinical manifestations and is confirmed by genetic testing. In our patient, a homozygous WFS1 gene mutation was identified, confirming the diagnosis of WS1. Advances in genetic screening now allow for earlier and more reliable detection of this disorder [12].

Currently, the management of WS remains largely symptomatic and requires a multidisciplinary approach involving endocrinologists, neurologists, urologists, ophthalmologists, and psychiatrists. Regular monitoring of endocrine, auditory, visual, and renal functions is essential to optimize quality of life and slow disease progression. Research is ongoing to develop targeted therapies focusing on endoplasmic reticulum stress and mitochondrial dysfunction, offering promising prospects for future treatments [1].

## Conclusions

This case highlights the importance of considering WS in patients with a combination of diabetes mellitus, optic atrophy, and unexplained urinary dysfunction, particularly in cases of consanguinity. Early recognition and appropriate management can improve patient outcomes and prevent severe complications. Further studies are needed to explore disease-modifying treatments targeting the endoplasmic reticulum stress pathway in WS.
